# [(2-Pyrid­yl)methanol-κ^2^ *N*,*O*]bis­(thio­cyanato-κ*N*)manganese(II)

**DOI:** 10.1107/S1600536810034483

**Published:** 2010-09-04

**Authors:** Qihe Gao, Qianqian Bao, Rong Rong

**Affiliations:** a Ordered Matter Science Research Center, College of Chemistry and Chemical Engineering, Southeast University, Nanjing 210096, People’s Republic of China; b Department of Chemistry, Key Laboratory of Medicinal Chemistry for Natural Resource, Ministry of Education, Yunnan University, Kunming 650091, People’s Republic of China

## Abstract

In the title complex, [Mn(NCS)_2_(C_6_H_7_NO)_2_], the Mn^II^ atom shows site symmetry 2. The distorted octa­hedral environment of Mn^II^ is defined by two N atoms [Mn—N = 2.217 (4) and 2.132 (5) Å] and one O atom [Mn—O 2.305 (4) Å]. There are inter­molecular O—H⋯S hydrogen bonds and inter­molecular π–π stacking inter­actions between adjacent (2-pyrid­yl)methano­late ligands [centroid–centroid distance = 3.5569 (7) Å], leading to a chain structure running along [100].

## Related literature

For background to metallacrowns, see: Mezei *et al.* (2007 ▶); Lah & Pecoraro (1989 ▶). For manganese clusters, see: Christou *et al.* (2000 ▶). For 2-(hy­droxy­meth­yl)pyridine, see: Shieh *et al.* (1997 ▶). For bond lengths and angles in related structures, see: Ito & Onaka (2004 ▶).
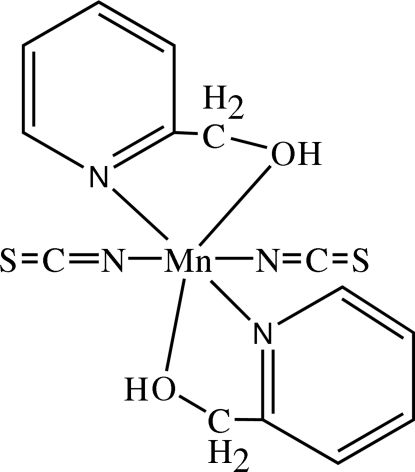


## Experimental

### 

#### Crystal data

[Mn(NCS)_2_(C_6_H_7_NO)_2_]
*M* *_r_* = 389.35
Orthorhombic, 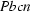

*a* = 11.4759 (12) Å
*b* = 8.398 (1) Å
*c* = 17.9451 (18) Å
*V* = 1729.5 (3) Å^3^
*Z* = 4
Mo *K*α radiation
μ = 1.02 mm^−1^
*T* = 298 K
0.48 × 0.45 × 0.40 mm

#### Data collection

Rigaku SCXmini CCD area-detector diffractometer
Absorption correction: multi-scan (*CrystalClear*; Rigaku, 2005 ▶) *T*_min_ = 0.641, *T*_max_ = 0.687
7935 measured reflections
1521 independent reflections
1214 reflections with *I* > 2σ(*I*)
*R*_int_ = 0.046

#### Refinement

*R*[*F* ^2^ > 2σ(*F* ^2^)] = 0.054
*wR*(*F* ^2^) = 0.135
*S* = 1.35
1521 reflections
105 parameters
H-atom parameters constrained
Δρ_max_ = 0.33 e Å^−3^
Δρ_min_ = −0.56 e Å^−3^

### 

Data collection: *CrystalClear* (Rigaku, 2005 ▶); cell refinement: *CrystalClear*; data reduction: *CrystalClear*; program(s) used to solve structure: *SHELXS97* (Sheldrick, 2008 ▶); program(s) used to refine structure: *SHELXL97* (Sheldrick, 2008 ▶); molecular graphics: *SHELXTL* (Sheldrick, 2008 ▶); software used to prepare material for publication: *SHELXL97*.

## Supplementary Material

Crystal structure: contains datablocks I, global. DOI: 10.1107/S1600536810034483/bg2365sup1.cif

Structure factors: contains datablocks I. DOI: 10.1107/S1600536810034483/bg2365Isup2.hkl

Additional supplementary materials:  crystallographic information; 3D view; checkCIF report

## Figures and Tables

**Table 1:** Hydrogen-bond geometry (Å, °)

*D*—H⋯*A*	*D*—H	H⋯*A*	*D*⋯*A*	*D*—H⋯*A*
O1—H1⋯S1^i^	0.82	2.49	3.297 (4)	167

Symmetry code: (i) 
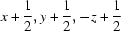
.

**Fig. 1.**
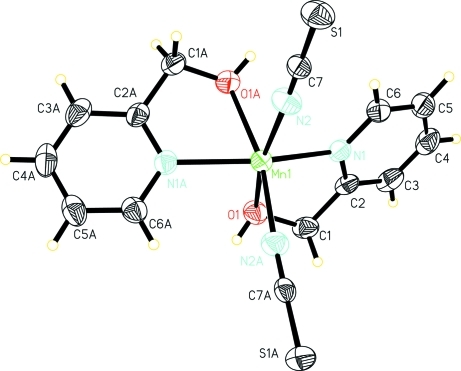
The molecular structure of the title compound with the atom-numbering scheme and all hydrogen atoms. Displacement ellipsoids are drawn at the 30% probability level. [Symmetry code A: 2 - x, -y, 1 - z]

**Fig. 2.**
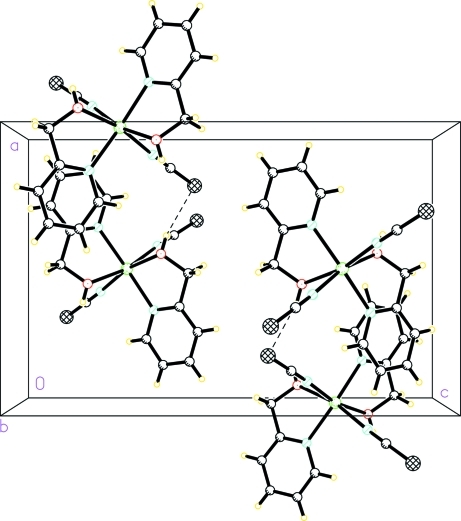
Crystal packing of the compound (1). Hydrogen bonds are shown as dashed lines.

## supplementary crystallographic information

### Comment

Some metallacrowns show diverse molecular architectures, selective recognition of ions and intramolecular magnetic exchange interactions (Lah & Pecoraro (1989); Mezei *et al.*2007). Among all metallacrowns, manganese clusters have been frequently investigated in recent years, because of their behavior in single molecule magnets(SMMs), which show magnetic hysteresis arising from slow magnetization reversal due to a high energy barrier (Christou *et al.* 2000). Pyridine derivatives with two ortho-substituents have recently been revised as an important supporting ligands of multiple metal-metal bonds and/or linear metal-metal bonded arrays which are composed by more than three metal atoms. 2-(hydroxymethyl)pyridine(Hhmp) is one of the preferred achelate ligands, because the alkoxide arm often supports ferromagnetic coupling between the metal atoms. Many nuclear manganese clusters based on hmp- have been obtained. It was clearly revealed that the Hhmp can function as a chelating ligand for a single manganese ion. In order to construct new structures based on manganese ions, we chose 2-(hydroxymethyl)pyridine (Hhmp) as a pyridine ligand (Shieh *et al.* 1997). Herein, we report a symmetric manganese complex, [Mn(Hhmp)_2_(SCN)_2_]. The compound presents a a Mn^II^ center on a two fold axis bisecting the distorted octahedral environment provided by one Hhmp, one SCN- and their symmetry related counterparts. A molecular view of the complex is shown in Fig.1. The Mn^II^ center is surrounded by two nitrogens from the SCN– anions (Mn—N2: 2.132 (5) Å), and two nitrogens and two oxygens from the chelating Hhmp ligands (Mn—N1: 2.217 (4), Mn—O1: 2.305 (4) Å) to form the distorted octahedral geometry. Distances and angles within the coordination environment of Mn^II^ are similar to those reported in Ito & Onaka (2004). Non bonding interactions include an intermolecular O—H···S hydrogen bond (Table 1) and a weak aromatic p···p stacking linking adjacent bpy ligand rings at (x, y, z) and (1/2-x, -1/2+y, z) (centroid-centroid distance: 3.557 (8)Å). These interactions define a 1D structure running along [100] (Fig.2).

### Experimental

All chemicals used (reagent grade) were commercially available. The reaction of MnCl_2_^.^4H_2_O, Hhmp, KSCN and triethylamine in a 2:5:5:1 molar ratio in MeCN/CH_3_CN (1:2, *v*/*v*) gave a dark solution with stirring. The resulting solution was continuously stirred for a moment, and then filtered. The filtrate was slowly evaporated at room temperature over several days, and dark quadrangle crystals suitable for X-ray analysis were obtained.

### Refinement

Positional parameters of all H atoms were calculated geometrically.

### Crystal data

**Table:**

[Mn(NCS)_2_(C_6_H_7_NO)_2_]	*F*(000) = 796
*M**_r_* = 389.35	*D*_x_ = 1.495 Mg m^−^^3^
Orthorhombic, *P**b**c**n*	Mo *K*α radiation, λ = 0.71073 Å
Hall symbol: -P 2n 2ab	Cell parameters from 3027 reflections
*a* = 11.4759 (12) Å	θ = 2.3–25.0°
*b* = 8.398 (1) Å	µ = 1.02 mm^−^^1^
*c* = 17.9451 (18) Å	*T* = 298 K
*V* = 1729.5 (3) Å^3^	Prism, dark brown
*Z* = 4	0.48 × 0.45 × 0.40 mm

### Data collection

**Table:**

Rigaku model name? CCD area-detector diffractometer	1521 independent reflections
Radiation source: fine-focus sealed tube	1214 reflections with *I* > 2σ(*I*)
graphite	*R*_int_ = 0.046
Detector resolution: 8.192 pixels mm^-1^	θ_max_ = 25.0°, θ_min_ = 2.3°
φ and ω scans	*h* = −8→13
Absorption correction: multi-scan (*CrystalClear*; Rigaku, 2005)	*k* = −8→9
*T*_min_ = 0.641, *T*_max_ = 0.687	*l* = −18→21
7935 measured reflections	

### Refinement

**Table:**

Refinement on *F*^2^	Primary atom site location: structure-invariant direct methods
Least-squares matrix: full	Secondary atom site location: difference Fourier map
*R*[*F*^2^ > 2σ(*F*^2^)] = 0.054	Hydrogen site location: inferred from neighbouring sites
*wR*(*F*^2^) = 0.135	H-atom parameters constrained
*S* = 1.35	*w* = 1/[σ^2^(*F*_o_^2^) + (0.0123*P*)^2^ + 5.1205*P*] where *P* = (*F*_o_^2^ + 2*F*_c_^2^)/3
1521 reflections	(Δ/σ)_max_ < 0.001
105 parameters	Δρ_max_ = 0.33 e Å^−^^3^
0 restraints	Δρ_min_ = −0.56 e Å^−^^3^

### Special details

**Table:**

Geometry. All e.s.d.'s (except the e.s.d. in the dihedral angle between two l.s. planes) are estimated using the full covariance matrix. The cell e.s.d.'s are taken into account individually in the estimation of e.s.d.'s in distances, angles and torsion angles; correlations between e.s.d.'s in cell parameters are only used when they are defined by crystal symmetry. An approximate (isotropic) treatment of cell e.s.d.'s is used for estimating e.s.d.'s involving l.s. planes.
Refinement. Refinement of *F*^2^ against ALL reflections. The weighted *R*-factor *wR* and goodness of fit *S* are based on *F*^2^, conventional *R*-factors *R* are based on *F*, with *F* set to zero for negative *F*^2^. The threshold expression of *F*^2^ > σ(*F*^2^) is used only for calculating *R*-factors(gt) *etc*. and is not relevant to the choice of reflections for refinement. *R*-factors based on *F*^2^ are statistically about twice as large as those based on *F*, and *R*- factors based on ALL data will be even larger.

### Fractional atomic coordinates and isotropic or equivalent isotropic displacement parameters (Å^2^)

**Table:**

	*x*	*y*	*z*	*U*_iso_*/*U*_eq_	
Mn1	0.5000	0.20604 (14)	0.2500	0.0459 (3)	
S1	0.30922 (15)	−0.1799 (2)	0.08535 (9)	0.0699 (5)	
N1	0.3448 (4)	0.2711 (5)	0.3170 (2)	0.0517 (12)	
N2	0.4118 (5)	0.0404 (6)	0.1798 (3)	0.0635 (14)	
O1	0.5552 (4)	0.3948 (5)	0.3363 (2)	0.0623 (11)	
H1	0.6229	0.3775	0.3485	0.093*	
C1	0.4838 (5)	0.3959 (8)	0.4006 (3)	0.0641 (17)	
H1A	0.4863	0.5002	0.4238	0.077*	
H1B	0.5124	0.3185	0.4363	0.077*	
C2	0.3599 (5)	0.3560 (7)	0.3791 (3)	0.0528 (14)	
C3	0.2673 (7)	0.4019 (8)	0.4232 (3)	0.0710 (19)	
H3	0.2802	0.4609	0.4663	0.085*	
C4	0.1568 (6)	0.3600 (9)	0.4032 (4)	0.077 (2)	
H4	0.0935	0.3889	0.4326	0.092*	
C5	0.1405 (6)	0.2744 (9)	0.3388 (4)	0.0731 (19)	
H5	0.0658	0.2465	0.3234	0.088*	
C6	0.2357 (5)	0.2308 (7)	0.2976 (3)	0.0600 (15)	
H6	0.2243	0.1710	0.2546	0.072*	
C7	0.3687 (5)	−0.0499 (7)	0.1405 (3)	0.0469 (13)	

### Atomic displacement parameters (Å^2^)

**Table:**

	*U*^11^	*U*^22^	*U*^33^	*U*^12^	*U*^13^	*U*^23^
Mn1	0.0442 (6)	0.0468 (6)	0.0466 (6)	0.000	0.0000 (5)	0.000
S1	0.0688 (10)	0.0746 (11)	0.0664 (10)	−0.0037 (9)	−0.0120 (8)	−0.0178 (9)
N1	0.053 (3)	0.055 (3)	0.047 (3)	0.005 (2)	0.003 (2)	0.006 (2)
N2	0.061 (3)	0.057 (3)	0.073 (3)	0.002 (3)	−0.010 (3)	−0.012 (3)
O1	0.056 (2)	0.074 (3)	0.057 (2)	−0.006 (2)	−0.003 (2)	−0.009 (2)
C1	0.068 (4)	0.078 (4)	0.046 (3)	0.014 (4)	−0.005 (3)	−0.007 (3)
C2	0.064 (4)	0.055 (3)	0.040 (3)	0.016 (3)	0.001 (3)	0.007 (3)
C3	0.091 (5)	0.076 (4)	0.047 (3)	0.023 (4)	0.007 (3)	0.007 (3)
C4	0.070 (5)	0.094 (5)	0.067 (4)	0.029 (4)	0.024 (4)	0.022 (4)
C5	0.052 (4)	0.090 (5)	0.077 (5)	0.014 (4)	0.007 (3)	0.024 (4)
C6	0.055 (4)	0.066 (4)	0.060 (4)	0.002 (3)	−0.001 (3)	0.011 (3)
C7	0.039 (3)	0.050 (3)	0.052 (3)	0.009 (3)	0.001 (3)	0.004 (3)

### Geometric parameters (Å, °)

**Table:**

Mn1—N2	2.132 (5)	C1—C2	1.511 (8)
Mn1—N2^i^	2.132 (5)	C1—H1A	0.9700
Mn1—N1^i^	2.217 (4)	C1—H1B	0.9700
Mn1—N1	2.217 (4)	C2—C3	1.379 (8)
Mn1—O1	2.305 (4)	C3—C4	1.365 (10)
Mn1—O1^i^	2.305 (4)	C3—H3	0.9300
S1—C7	1.624 (6)	C4—C5	1.373 (10)
N1—C2	1.335 (7)	C4—H4	0.9300
N1—C6	1.343 (7)	C5—C6	1.368 (8)
N2—C7	1.148 (7)	C5—H5	0.9300
O1—C1	1.415 (6)	C6—H6	0.9300
O1—H1	0.8200		
			
N2—Mn1—N2^i^	98.5 (3)	O1—C1—C2	109.6 (4)
N2—Mn1—N1^i^	102.81 (18)	O1—C1—H1A	109.7
N2^i^—Mn1—N1^i^	95.73 (19)	C2—C1—H1A	109.7
N2—Mn1—N1	95.73 (19)	O1—C1—H1B	109.7
N2^i^—Mn1—N1	102.81 (18)	C2—C1—H1B	109.7
N1^i^—Mn1—N1	151.5 (2)	H1A—C1—H1B	108.2
N2—Mn1—O1	167.46 (18)	N1—C2—C3	121.9 (6)
N2^i^—Mn1—O1	85.49 (17)	N1—C2—C1	117.0 (5)
N1^i^—Mn1—O1	88.50 (15)	C3—C2—C1	121.1 (6)
N1—Mn1—O1	71.76 (16)	C4—C3—C2	119.5 (6)
N2—Mn1—O1^i^	85.49 (17)	C4—C3—H3	120.3
N2^i^—Mn1—O1^i^	167.46 (18)	C2—C3—H3	120.3
N1^i^—Mn1—O1^i^	71.76 (16)	C3—C4—C5	118.9 (6)
N1—Mn1—O1^i^	88.50 (15)	C3—C4—H4	120.5
O1—Mn1—O1^i^	93.1 (2)	C5—C4—H4	120.5
C2—N1—C6	118.1 (5)	C6—C5—C4	119.0 (7)
C2—N1—Mn1	118.7 (4)	C6—C5—H5	120.5
C6—N1—Mn1	123.2 (4)	C4—C5—H5	120.5
C7—N2—Mn1	177.1 (5)	N1—C6—C5	122.6 (6)
C1—O1—Mn1	113.1 (3)	N1—C6—H6	118.7
C1—O1—H1	109.5	C5—C6—H6	118.7
Mn1—O1—H1	108.5	N2—C7—S1	179.0 (5)
			
N2—Mn1—N1—C2	168.6 (4)	Mn1—O1—C1—C2	−34.4 (6)
N2^i^—Mn1—N1—C2	68.5 (4)	C6—N1—C2—C3	0.2 (8)
N1^i^—Mn1—N1—C2	−60.7 (4)	Mn1—N1—C2—C3	179.4 (4)
O1—Mn1—N1—C2	−12.3 (4)	C6—N1—C2—C1	178.4 (5)
O1^i^—Mn1—N1—C2	−106.0 (4)	Mn1—N1—C2—C1	−2.3 (7)
N2—Mn1—N1—C6	−12.1 (5)	O1—C1—C2—N1	24.8 (7)
N2^i^—Mn1—N1—C6	−112.3 (4)	O1—C1—C2—C3	−156.9 (5)
N1^i^—Mn1—N1—C6	118.5 (4)	N1—C2—C3—C4	0.0 (9)
O1—Mn1—N1—C6	166.9 (5)	C1—C2—C3—C4	−178.2 (6)
O1^i^—Mn1—N1—C6	73.2 (4)	C2—C3—C4—C5	−0.8 (10)
N2—Mn1—O1—C1	30.2 (10)	C3—C4—C5—C6	1.4 (10)
N2^i^—Mn1—O1—C1	−79.2 (4)	C2—N1—C6—C5	0.5 (9)
N1^i^—Mn1—O1—C1	−175.1 (4)	Mn1—N1—C6—C5	−178.7 (5)
N1—Mn1—O1—C1	25.9 (4)	C4—C5—C6—N1	−1.3 (10)
O1^i^—Mn1—O1—C1	113.3 (4)		

Symmetry codes: (i) −*x*+1, *y*, −*z*+1/2.

### Hydrogen-bond geometry (Å, °)

**Table:**

*D*—H···*A*	*D*—H	H···*A*	*D*···*A*	*D*—H···*A*
O1—H1···S1^ii^	0.82	2.49	3.297 (4)	167

Symmetry codes: (ii) *x*+1/2, *y*+1/2, −*z*+1/2.
